# Unincorporation in counties as a political determinant of health: An exploration of five states

**DOI:** 10.1016/j.ssmph.2024.101728

**Published:** 2024-11-19

**Authors:** Cristina Gomez-Vidal, Ashley N. Palmer, Katherine Kitchens, G. Allen Ratliff, Genevieve Graaf

**Affiliations:** a School of Social Welfare, University of California, Berkeley 120 Haviland Hall, Berkeley, CA, 94709, USA; b Department of Social Work, Harris College of Nursing & Health Sciences, Texas Christian University, 2800 South University Drive, Fort Worth, TX, 76109, USA; c School of Social Work, University of Texas at Arlington, 501 W. Mitchell Street, Arlington, TX, 76019-0129, USA; d School of Social Work, University of Nevada, 1664 N. Virginia Street, Reno, NV, 89557, USA

## Abstract

Local government policies and practices shape the context of the places that can alter a population's life chances through socioeconomic factors, built environments, and healthcare access. County governments, one of the most ubiquitous U.S. political structures, impact health outcomes within their jurisdiction through multiple policy levers and pathways. By identifying which political determinants within counties are associated with variation in life expectancy and premature death, we can better intervene on modifiable factors. One overlooked political determinant from public health studies is the county's responsibility as the primary local government for approximately a third of the U.S. population and 93% of land in unincorporated areas. To conduct an ecological study and assess associations between county population health and county responsibility for unincorporated areas and populations, we created and tested two county indicators of unincorporation. Multilevel multivariable regression results showed that larger proportions of unincorporated land within a county are associated with lower average county life expectancy. More testing of the measurement is needed to understand the relationship between unincorporation, county government, and population health outcomes.

## Highlights

• County-level non-health factors are significantly associated with poor health outcomes.
• Two indices of Unincorporation were piloted to examine county health outcomes.
• Larger proportions of county unincorporated land are associated with lower life expectancy.

## 1 Introduction

The impact of political determinants in shaping health outcomes is often obscured in discourses on individual diagnoses, health epidemics, and stories of birth and death. Examining aggregates of population health can elucidate how the political architecture of where and how we live constrains or fosters our well-being (Muntaner et al., 2012). Life expectancy as a measure of population health has been a benchmark by which political regimes have evaluated how their social, political, and economic infrastructure supports a population's health within its jurisdiction (Avendano & Kawachi, 2014; Ho & Hendi, 2018). This benchmark is particularly relevant considering that, in recent decades, the mortality rate in the United States has exceeded that of most other high-income countries (Murphy et al., 2021; Woolf & Aron, 2013).

Comparing political structures across different levels of governance can illuminate factors contributing to health disparities, even among wealthy nations. Political determinants shape places through numerous governance structures, systems, policies, and practices enacted by governments at local, state, and federal levels (Dawes, 2020; Kickbusch, 2013, 2015). The cumulative interplay of these determinants constructs vital health factors, including healthcare infrastructure, environmental risks, and residential lifestyles through direct and indirect pathways (Dawes, 2020; Hood et al., 2016). These health factors, in turn, influence health outcomes, including life expectancy and premature death. Local political determinants of health are uniquely situated to influence health outcomes, due to their proximal role in shaping place and opportunities that support residents’ health, such as access to healthcare services, healthy food options, and safe living environments (Kickbusch, 2013).

## 2 Background

### 2.1 Political determinants of health and local governments

Political determinants that operate through local government structures (i.e., municipalities and cities, counties, boroughs, parishes, townships, and others) are pivotal to creating healthy places and people (Van Vliet-Brown et al., 2018). Local political decisions shape community socioeconomic and physical development, healthcare access, infrastructure, and housing qualities that impact individual and family health and well-being (Dattani et al., 2023; Northridge et al., 2010; Onofrei et al., 2021; Pelletier et al., 2021; Riley, 2018; Roof & Oleru, 2008; Van Vliet-Brown et al., 2018). For example, life expectancy steadily increased in the Global North and West during the 20th century due to national and local government investments in water, sanitation, nutrition, and public health (Cutler et al., 2006). Conversely, the underdevelopment of these infrastructures in the Global South is cited as a reason for higher mortality rates and an area of needed policy intervention (Rahman et al., 2022).

Similarly, local governments such as cities and counties contribute to health through decision-making that shapes residents' exposure to health threats and access to health-protective resources (Hallas et al., 2021; Hamad et al., 2022). Variations and contradictions in local government responses to the COVID-19 pandemic, such as flexibility and willingness of local governments to respond to the public health crisis (Unruh et al., 2022), imposition of vaccine mandates (Karaivanov et al., 2022), and health information provision (Hansen et al., 2021) were found to contribute to disparities in frequency and severity of COVID-19 outcomes. Furthermore, findings show that smaller and less resourced local governments have a lower capacity to meet emergency needs during a weather-related emergency, exacerbating social inequalities in a community's ability to respond or recover (Dzigbede et al., 2020). Given the importance of government decision-making on population health, research on factors that contribute to local government's capacity to respond to residents' needs can better explain how health disparities emerge between jurisdictions.

County governments and their equivalents (e.g., boroughs in Alaska and parishes in Louisiana) impact health factors and outcomes through the distribution of goods, services, revenues, and procedural (i.e., who, how, and when) decision-making (Anderson, 2008, 2010; Gomez-Vidal & Gomez, 2021; Rivera et al., 2022). Evidence demonstrates that county government decision-making on the distribution of transportation, energy production, and expenditures related to infrastructure and service provision is associated with resident life expectancy (Erwin et al., 2012; Leider et al., 2020; McCullough et al., 2019, 2020) and premature death (Mays & Smith, 2011; Ronzio et al., 2004). Furthermore, comparing the type of county government structure yielded differences in life expectancy (Knepper, 2012). These differences suggest that reformed county governance, compared to traditional government structures, theorized as being more accountable to local constituents than traditional governance, is associated with better health outcomes (Knepper, 2012).

Although there are multiple definitions for government capacity, it is generally defined as the ability to perform functions effectively and efficiently (Reddy et al., 2015). This study focuses on a county government's capacity to shape the social determinants impacting residents' health through its material, political, and human resources (Benton, 2002, Martin, Kousky, French, & Donoghoe, 2023, Piña & Avellaneda, 2017). Factors that impact a county's capacity to meet social determinants of health include financial, personnel, and expertise associated with significant population demand and expectations for infrastructure, rising costs and economies of scale when providing municipal services Benton, 2002, Benton et al., 2007, Benton et al., 2008, Chen & Bartle, 2017, and expertise and resources to meet complex challenges such as housing, and the built and natural environment (Ahmadu & Nukpezah, 2022, Anderson, 2008, Dzigbede, Gehl, & Willoughby, 2020, Martin, Kousky, French, & Donoghoe, 2023, Schneider, 2018). While counties play an essential role in promoting social determinants of health for all county residents through the provision of essential road infrastructure and health and human services, unincorporated residents of a county (living outside of cities) rely solely on the county to act as a local government (Anderson, 2008; Gomez-Vidal & Gomez, 2021). A commonly overlooked factor in population health studies is how a county government's ability to meet population needs is impacted by its responsibility to unincorporated areas and populations under its jurisdiction.

### 2.2 Unincorporation as a political determinant of health

Over one-third of the United States population live in unincorporated areas (Cohen et al., 2015), defined as territories outside incorporated city and municipal jurisdictional boundaries (Flegal et al., 2013). Unincorporated area populations vary in characteristics and live in disparate locations, ranging from isolated dwellings in perceived hard-to-reach rural areas to densely populated urban suburbs outside city borders (Anderson, 2010). Regardless of size, longevity, or historicity, a place that has not been legally incorporated through a state's process does not have the authority to form a local government and has the political status of an unincorporated place in most states.

Residents of incorporated municipalities have an additional and more proximal level of governance at the municipal level (Anderson, 2008, 2010; Gomez-Vidal & Gomez, 2021; Purifoy, 2020). This incorporation includes dedicated city councils, mayors, or city departments dedicated to shaping housing, services, economic development, amenities or disamenities, tax and revenue-generating tools, and opportunities that can foster healthy environments for those within their jurisdiction. This is all in in addition to any resource allocation or collaboration from county governments. Municipal governments tend to have greater capacity, resources, and expertise tailored to serving as a local government than counties whose primary responsibility is as an arm of the state (Anderson, 2008, Lobao & Kraybill, 2005, Pemberton, 2022). Unincorporated area residents lack the local proximal tier of representation through a city government and so depend on county policymakers to be responsible for conditions in unincorporated areas (Anderson, 2008). Thus, unincorporated areas and populations require more from a county government than residents in cities and towns (Lang, Blakely, & Gough, 2005, Munroe, 2010). This relationship suggests that the greater the proportion of unincorporated areas and population for which a county is responsible, the greater a county's capacity as a local government is taxed based on the demand for services, resources, and personnel.

While having broad responsibilities and dedicated resource capacity to serve the functions associated with being an arm of the state, the ability of county governments to meet the infrastructure and service needs of local residents may be constrained due to limited authority and resources when compared with municipal government (Anderson, 2008, Gomez-Vidal and Gomez, 2021, Pemberton, 2022). Typically, county governments, compared to cities, have less local governance authority, different or fewer fiscal or policy tools available, and reduced flexibility to meet community development and service needs (Anderson, 2008, 2010; Flegal et al., 2013; Mattiuzzi & Weir, 2020; Pemberton, 2022). Some states restrict county land use authority, limiting a county's ability to support healthy built environments (Durst, 2016). Further, federal grants and other financial mechanisms that provide local government support to meet municipal needs and promote economic and community development are often targeted for delivery via cities or towns rather than counties (Anderson, 2008; Rivera, 2023; Rivera et al., 2022). When Anderson (2008) compared city government capacity to meet city residents needs (e.g., adequate housing mobility, habitability, political participation) with county government capacity to provide for unincorporated community residents, they found that counties were weaker than cities in providing resources and services to shape these critical social determinants. Limited county capacity to meet resident needs can leave unincorporated and populations more vulnerable to poor environmental and social conditions that negatively impact health, which are then reflected in lower county health outcomes.

### 2.3 Study aims

Given that counties are the primary responsible government for residents of unincorporated areas, we explore how the population and size of these areas within a county are associated with population health indicators. Along with state constraints on counties, we hypothesize that unincorporated area size and population may also strain county government capacity to meet resident needs based on area size and population. Counties with sizeable unincorporated areas (i.e., most counties in the United States) may have large expanses of territory with geographically dispersed populations to serve, hampering a county's ability to service these perceived hard-to-reach areas (Mattson, 2020). Additionally, when unincorporated areas are located in proximal distance to incorporated areas, residents' demands and expectations are often similar to those of municipal resident neighbors. If counties are to provide residents with connection to nearby municipal services, it will require costly infrastructure, government collaboration, and complex planning (Benton, 2002, Benton et al., 2008, Lang, Blakely, & Gough, 2005, Mohr, Deller, & Halstead, 2010). Importantly, it has been theorized that low-income unincorporated areas with marginalized populations have poor environmental conditions and greater exposure to unwanted land uses (i.e., polluting industries, waste management) due to limited political representation and more lax zoning (Anderson, 2008; Bullard, 2000; Gomez-Vidal & Gomez, 2021; Marsh et al., 2010; Purifoy, 2021; Schively, 2007). Evidence demonstrates that many unincorporated areas disproportionately house environmental hazards (Arata, 2016; Bullard, 2000; Bullard & Johnson, 2000) and are more likely to have poor water quality (Adams & Perkin, 1985; Korc & Ford, 2012; London et al., 2021; Pemberton, 2022) and waste disposal (Anderson, 2008; Pemberton, 2022). Counties with larger populations exposed to these risks or larger proportions of their land filled with these risks may also have poorer population health outcomes. This study seeks to answer the following questions: Are higher proportions of unincorporated areas in a county associated with county health outcomes? Are higher proportions of unincorporated area populations in a county associated with county health outcomes? To explore the relationship between county levels of unincorporation and county population health, this ecological study estimates the relationship between population and area measures of county unincorporation and county population life expectancy and premature death.

## 3 Methods

This exploratory ecological study drew upon data from the County Health Rankings and Roadmaps (CHRR) (Hood et al., 2016) public-use analytic files from 2019, 2020, and 2021, U.S. Census Bureau Geography TIGER/Line place, U.S. Census Annual Estimates of Resident Populations for Incorporated Places in the United States, and American Community Survey (ACS) Demographic and Housing Estimates County Total population for 2019. U.S. Census Bureau data sets were selected to create indicators measuring the unincorporated proportion of a county's population and area to examine relationships between the proportion of a county's population and unincorporated area with distal health outcomes in each county (U.S. Census, 2021). We observed the Unincorporated area and population at the beginning of our study period on the assumption that this metric is unlikely to change meaningfully in the latter two years of the study period (U.S. Census, 2023).^2^ Three years of data was used both to increase sample size, and associated statistical power, and to account for minor fluctuations in variables over time. Analysis used county-level health outcome measures from the CHRR data from five states: California, Georgia, Nebraska, New York, and Texas. The sample was limited to these states because the development of county unincorporation measures was unprecedented and, as a result, time intensive. Additionally, this study sought to pilot a new index by assessing its predictive power concerning county-level health outcomes. Limiting the sample of states allowed for changes in the measures if needed upon completion of the pilot study.

### 3.1 Sample

California, Georgia, Nebraska, New York, and Texas were selected due to factors related to state geographic, demographic, and government structure diversity. First, California and Texas are the two most populous states in the United States and are ranked third and second, respectively, in total land area. Moreover, these states differ dramatically—not only in the size and number of counties (California has 58 counties compared to Texas's 254 counties) but also in social, economic, and political contexts. New York has a northeastern model of townships and a large population disproportionately dispersed geographically, making it comparable and different to California and Texas. Nebraska was chosen due to its small population related to other medium-large area states and its inconsistent use of townships and median-level jurisdictions (i.e., about a third of Nebraska counties include townships, while the rest do not). Georgia was included due to its high level of county government autonomy (NACO Research, 2022). The sample included 626 counties in five states (CA = 58, TX = 254, GA = 159, NY = 62, NE = 93) observed over three years, totaling 1878 county/year observations. Creating and piloting the index in these five states provides a rich opportunity to compare effects in diverse physical and political settings.

### 3.2 Data

CHRR analytic files for 2019, 2020, and 2021 were downloaded separately, restricted to counties for the five sample states, and appended. Additional county-level control variables were downloaded from PolicyMap, a public-use analytic and mapping platform. U.S. Census Bureau Geography TIGER/Line place shape files and the American Community Survey (ACS) Demographic and Housing Estimates County total population data for 2019 (US Census Bureau, 2022b, US Census Bureau, 2022a) were used to create two pilot unincorporated indicators – the proportion of population living in unincorporated areas within counties (Population Index) and the proportion of unincorporated surface area within counties (Area Index). The U.S. Census Bureau Geography TIGER/Line provides spatial files with legal boundaries for places in the U.S. that can be linked to census data. The ACS provides annual estimates of national population characteristics. Detailed information about ACS methodology, sampling, and weighting procedures are described in publicly available documentation (ACS and PRCS Design and Methodology (Version 3.0), 2022). Both unincorporated status indicators and the PolicyMap data were merged into the aggregate CHRR dataset using the five-digit FIPS code from Census data.

### 3.3 Measures

**Independent variable**. Our independent variables were the two pilot unincorporated status indicators. First, the “Unincorporated Area Index” is the proportion of the square mileage of unincorporated areas within a county (unincorporated area/total county area). U.S. Census Bureau 2019 TIGER/line shapefiles for counties and cities were spatially joined to identify and remove incorporated areas from each county using GIS in R 4.2.0 to identify remainder of unincorporated area (US Census Bureau, 2022b). The second measure, the “Unincorporated Population Index,” represents the proportion of the population living in unincorporated areas within a county (population living in unincorporated areas/total county population). U.S. Census Bureau 2019 population data from the ACS (US Census Bureau, 2022a) were utilized to identify and remove incorporated populations from total county populations using Python 3.7.11. As proportional measures, values for both variables range from 0 to 1, with 1 referring to a completely unincorporated county, where a 0 refers to a completely incorporated county. A simple correlation assessment of the population index and the area index indicates a moderate positive correlation (0.42) between the measures. This suggests that these indicators capture distinctly different, though related, constructs. For more details on the construction of the piloted indicators, see Ratliff et al., which is under review.

**Dependent Variable.** Two county measures of health outcomes were examined concerning the proportions of unincorporated population and area within counties: life expectancy and premature death. CHRR data sources for each health outcome variable are listed in Appendix A.

*Life expectancy*. Data for the CHRR life expectancy measure comes from the National Center for Health Statistics (NCHS)- Mortality Files (2019-2015-2017) and reflects the average number of years from birth that one may expect to live (age-adjusted). There were 54 missing observations (i.e., 18 counties missing all three annual observations) for this continuous variable; 51 were in Texas.

*Premature death*. The CHRR premature death variable data comes from the National Center for Health Statistics (NCHS) - Mortality Files. The measure of premature death utilized in this study was Years of Potential Life Lost (YPLL), an age-adjusted measure that reflects the years of potential life lost before age 75 per 100,000 within a given county. For this measure, a higher number reflects higher rates of premature death. There were 31 missing observations for this continuous variable, all in Texas (i.e., ten counties missing all three observations, one county missing one observation).

**Covariates**. Several control variables were selected for this analysis, drawing from the Social Determinants of Health Framework (Office of Disease Prevention and Health Promotion, 2015) and the County Health Rankings Model (CHRR, 2023). From the CHRR data, this study utilized the following county-level variables: 1) *Median household income* (the income where half of households in a county earn more and half of households earn less); 2) *Unemployment rate* (the proportion of population ages 16+ unemployed but seeking work); 3) *High school completion* (the percentage of adults ages 25 and over with a high school diploma or equivalent, only available in the 2021 CHRR data); 4) *Rural population*^3^ (the proportion of population living in rural areas); and 5) *Uninsured rate* (proportion of population under age 65 without health insurance). CHRR data sources for each control variable are listed in Appendix A. Additional county contextual variables from the PolicyMap database (*PolicyMap | Mapping, Analytics, and Data Visualization*, n.d) were included in the analyses. County levels of racialized residential segregation were accounted for using Theil's H index (Iceland, 2004), drawn by PolicyMap from the U.S. Census Bureau 2010 Decennial Census (US Census Bureau, n.d.). This index is continuous and reflects the level of racial segregation by measuring the evenness of distribution of diversity across multiple racial/ethnic categories within a county; values range from 0 = more uniform racial and ethnic distributions to 1 = less uniform racial and ethnic distributions.

Additionally, the accessibility of health care services in a county was approximated using PolicyMap's county *primary care physician ratio* variable. PolicyMap's measure was created using 2016 Health and Resources Service Administration (HRSA) data (HRSA, 2016), indicating the number of practicing primary care physicians per 1000 people.

To account for varying county administrative burdens associated with sparseness (i.e., counties with smaller total populations and larger areas to cover or density (i.e., counties with large total populations concentrated in small areas), two continuous variables were also created using the 2019 U.S. Census Bureau data for area and population in states and counties. One variable measured the county's *population relative to the state population* (county population/state population). A second measure represented the *surface area within a county relative to the total surface area of the state* (county surface area/state surface area).

### 3.4 Analytic procedure

Univariate analysis for each was conducted to assess the distribution of each variable, examining the distribution and measures of centrality and dispersion for each. For all variables, less than 10% of observations had missing data, and these were handled using listwise deletion. Bivariate analysis assessed the independent relationship between each predictor and control variable and each outcome variable. Multilevel linear models clustered annual observations within counties, controlled for community-level characteristics for each year, and employed state and year fixed effects and county and state random effects. Two separate models were constructed for each health outcome, resulting in four total models. One set of models assessed relationships between the Unincorporated Population Index and Unincorporated Area Index and county-level measures of life expectancy. The second set of models assessed associations between the Unincorporated Population Index and Unincorporated Area Index and county-level measures of premature death. Variance inflation factors (VIFs) were estimated for each variable in each of our models. With the exception of with the exception of the *Uninsured rate* variable—all VIFs were under 5. The VIF for *Uninsured rate* ranged between 7 and 9.

Sensitivity analysis and robustness checks included examining interactions between the unincorporated population and area variables and residential segregation and rural population measures to assess for a differential relationship between our predictor variable and health outcomes that may exist in residentially segregated counties or more rural counties. We examined the statistical fit of each model using the Bayesian information criterion (BIC) statistic, which indicates model fit and privileges with sensitivity analyses (Browne & Draper, 2006). The models presented here represent a balance between statistical fit and relevance of the models using known covariates identified as social determinants of health and the study theory and research questions. All analyses were conducted using Stata 16.0.

## 4 Results

Table 1^4^ reflects the characteristics of our sample of counties, combined and disaggregated by state. In 2019, the average proportion of a county's population living in unincorporated areas was 0.49 or 49% of the population (*S.D.* = 0.24). Across the sample, on average, 93% of a county's total surface area was unincorporated (*SD* = 0.17). The average life expectancy within the sample across the three years of data available was 77.87 years (*SD* = 2.79). The average YPLL for the aggregate sample was 8144.98 years per 100,000 residents in a given county (*SD* = 2600.41).

**Table 1:** Sample Characteristics Individual state values are a summary of values across counties and do not reflect total overall population values. See Appendix B for variable descriptions.

	Combined		California		Texas		Georgia		Nebraska		New York	
	M	SD	M	SD	M	SD	M	SD	M	SD	M	SD
Indices												
Population	0.49	0.24	0.42	0.29	0.43	0.23	0.62	0.22	0.4	0.18	0.6	0.22
Area	0.93	0.17	0.91	0.17	0.95	0.12	0.89	0.23	0.98	0.05	0.88	0.27
Health Outcomes												
Life expectancy	77.87	2.79	80.31	3.31	77.44	2.42	76.18	2.33	79.37	2.15	79.61	1.71
Premature death (YPLL)	8144.98	2600.41	6991.45	4111.80	8471.51	2283.50	9461.10	2075.54	6702.99	2088.22	6410.74	1040.26

Controls	M	SD	M	SD	M	SD	M	SD	M	SD	M	SD
Rural population	0.56	0.33	0.29	0.29	0.56	0.32	0.61	0.29	0.75	0.31	0.44	0.28
Residential segregation	0.4	0.12	0.27	0.07	0.38	0.09	0.41	0.11	0.54	0.11	0.35	0.08
High school completion	0.84	0.08	0.85	0.07	0.81	0.08	0.83	0.06	0.92	0.04	0.89	0.04
Unemployment rate	0.04	0.02	0.05	0.03	0.04	0.01	0.04	0.01	0.03	0.01	0.05	0.01
Median household income	53,530.85	14,766.84	67,448.78	20,677.60	51,869.04	12,732.70	47,801.85	13,734.52	54,027.06	7720.32	61,266.68	15,233.20
Uninsured proportion	0.16	0.06	0.08	0.02	0.21	0.04	0.17	0.03	0.11	0.03	0.01	0.01
PCP ratio (per 1000 persons)	0.47	0.33	0.69	0.32	0.38	0.27	0.43	0.39	0.53	0.45	0.62	0.39
County area proportion	0.01	0.01	0.02	0.02	0	0	0.01	0	0.01	0.01	0.02	0.01
County population proportion	0.01	0.02	0.02	0.04	0	0.01	0.01	0.01	0.01	0.04	0.02	0.03

95% confidence intervals in brackets *∗p* < .05. *∗∗p* < .01*. ∗∗∗p* < .001.

Regarding other sociodemographic characteristics of counties, on average, 56% of a county's population lived in a rural area (*SD* = 0.33). Residential racial segregation, as measured by the Theil-H index, was about 0.40 on average (*SD* = 0.12. The counties in our dataset had moderate levels of racial residential segregation, based on this metric where 0 = more uniform racial and ethnic distributions and 1 = less uniform racial and ethnic distributions. Across all counties, over 84% of county residents over the age of 25 had a high school diploma. On average, 4% of the county population ages 16 and older were unemployed but seeking work. The average annual median household income across our sample of counties was $53,530.85 (*S.D.* = $14,766.84). Approximately 16% of people under the age of 65 lacked health insurance (*SD* = 0.06). Overall, the PCP ratio across the counties in our sample was 0.47 (SD = 0.33) per 1000 residents. As seen in Table 1, the proportion of the county population relative to the state's total population and the county surface area relative to the state's total surface area is roughly the same within a given state. For the whole sample, counties had, on average, about 0.08% of the state population (SD = 0.023) living within them and represented approximately the same proportion of the area of the whole state (SD = 0.009).

Table 2 displays the bivariate associations between the unincorporated indicators and the sociodemographic control variables and health outcomes included in this study. A higher proportion of a county population living in unincorporated areas is associated with lower life expectancy (β = −2.14; CI: −2.67, −1.60) and slightly higher premature death rates (β = 2343.25; CI: 1850.88, 2835.62). higher proportion of unincorporated county surface area is associated with lower life expectancy (β = −2.78; CI: −3.49, −2.07) and higher rates of premature death (β = 1838.03; CI: 1166.75, 2509.32).

**Table 2:** Unincorporation associations with county characteristics and health factors.

	% Unincorporated Population			% Unincorporated Area		
	*b*	95% CI		*b*	95% CI	
		LL	UL		LL	UL
Health Outcomes						
Life expectancy	−0.02^c^	−0.02	−0.01	−0.01^c^	−0.01	−0.01
Premature death	0.00^c^	0.00	0.00	0.00^c^	0.00	0.00
Controls						
Rural population	0.32^c^	0.29	0.35	0.21^c^	0.19	0.24
Residential segregation	0.18^c^	0.09	0.27	0.35^c^	0.29	0.42
High school completion	0.31^c^	0.18	0.45	−0.11^a^	0.21	−0.01
Unemployment rate	1.85^c^	1.14	2.56	−0.08	−0.59	0.44
Median household income	−0.00^b^	−0.00	−0.00	−0.00^c^	−0.00	−0.00
Population under age 65 without health insurance	−0.28^b^	−0.45	−0.10	0.27^c^	0.15	0.40
Primary Care Physician ratio	−0.20^c^	−0.23	−0.17	−0.14^c^	−0.16	−0.12
County proportion of state population	−2.56^c^	−3.02	−2.10	−3.61^c^	−3.91	−3.31
County proportion of state area	1.19	−0.01	2.40	2.19^c^	1.33	3.05
Year	0.00	−0.01	0.01	0.00	−0.01	0.01
State						
California	−0.07^c^	−0.11	−0.04	−0.02	−0.05	0.01
Georgia	0.18^c^	0.15	0.20	−0.05^c^	−0.07	−0.03
Nebraska	−0.06^c^	−0.09	−0.03	0.05^c^	0,03	0.07
New York	0.13^c^	0.09	0.16	−0.05^c^	−0.08	−0.03
Texas	−0.10^c^	−0.13	−0.08	0.03^c^	0.02	0.05

***Notes***. 95% confidence intervals in brackets.

a *p* < .05.

b *p* < .01*.*

c *p* < .001.

Table 3 shows adjusted associations between county-level measures of unincorporation and county health outcomes. Each column in Table 3 represents findings from one of each of our four models. Column 1 shows model results for life expectancy regressed on the population index; Column 2 shows model results for life expectancy regressed on the area index. Column 3 shows model results for premature death regressed on the population index, and Column 4 shows model results for premature death regressed on the area index. Holding sociodemographic and structural factors constant, the proportion of the county surface area that is unincorporated was associated with mean life expectancy in the county (β = −1.82; CI: −2.93, −0.70) but not county rates of premature death. Further, when controlling for all other county sociodemographic and structural factors, the proportion of county populations living in unincorporated areas was found not to be associated with county rates of premature death or mean county left expectancy. County characteristics related to both life expectancy and premature death include median household income, unemployment, and high school completion rate. Residential segregation and primary care physician ratios were also associated with premature death.

**Table 3:** Associations between unincorporation and county level health outcomes.

	Life Expectancy				Premature Death			
	Population		Area		Population		Area	
	β	95% CI	β	95% CI	β	95% CI	β	95% CI
Index	−0.27	[-1.19,0.66]	−1.82^b^	[-2.93,-0.70]	588.45	[-282.68,1459.58]	−218.4	[-1290.47,853.68]
Population without health insurance	2.49	[-1.62,6.61]	2.49	[-1.62,6.60]	−1278.49	[-5504.48,2947.51]	−1231.79	[-5459.39,2995.81]
Primary Care Physician ratio	0.15	[-0.53,0.82}	0.06	[-0.610.73]	−632.14^a^	−1264.35,0.07]	−714.50^a^	[-1342.82,-86.18]
Rural population	0.22	[-0.62,1.06]	0.34	[-0.45,1.12]	473.35	[-320.67,1267.38]	691.47	[-59.99,1442.93]
Residential segregation	−2.04	[-4.44,0.15]	−2.12	[-4.38,0.15]	4998.96^c^	[2844.21,7153.71]	4828.82^c^	[2683.82,6973.82]
High school completion	−3.46^a^	[-6.83,-0.10]	−3.79^a^	[-7.08,-0.49]	4672.56^b^	[1467.44,7877.68]	5011.69^b^	[1845.62,8177.76]
Median household income	0.00^c^	[0.00,0.00]	0.00^c^	[0.00,0.00]	−0.05^c^	[-0.06,-0.04]	−0.05∗∗∗	[-0.06,-0.04]
Unemployment rate	−14.54^b^	[-22.87,-6.20]	−14.83^c^	[-23.12,-6.54]	9537.45^a^	[678.30,18396.59]	9979.00^a^	[1140.52,18817.47]
Year	−0.16^c^	[-0.22,-0.10]	−0.16^c^	[-0.22,-0.10]	192.58^c^	[127.39,257.77]	193.00^c^	[127.79,258.22]
County proportion of state population	11.02^b^	[3.22,18.82]	5.95	[-2.40,14.29]	−2278.94	[-9714.25,5156.37]	−3425.34	[-11459.82,4609.13]
County proportion of state area	5.29	[-15.44,26.02]	11.74	[-9.20,32.68]	−14733.66	[-34457.37,4990.06]	−13018.03	[-33134.24,7098.17]
State (Texas is reference)								
California	2.22^c^	[1.37,3.07]	2.21^c^	[1.37,3.06]	−123.52	[-946.31,699.26]	−95.11	[-917.90,727.68]
Georgia	−0.73^b^	[-1.22,-0.24]	−0.89^c^	[-1.34,-0.43]	328.19	[-139.39,795.77]	428.08	[-14.11,870.28]
Nebraska	2.32^c^	[1.51,3.13]	2.43^c^	[1.63,3.22]	−2678.49^c^	[-3454.50,-1902.47]	−2737.92^c^	[-3509.63,-1966.21]
New York	2.19^c^	[1.32,3.05]	2.07^c^	[1.22,2.92]	−1810.24^c^	[-2659.74,-960.74]	−1715.87^c^	[-2553.72,-878.03]

N	1779		1779		1806		1806	
AIC	5715.67		5705.85		31003.07		31004.66	
BIC	5814.38		5804.56		31102.05		31103.64	

a p < .05.

b p < .01.

c p < .001.

## 5 Discussion and conclusion

### 5.1 Discussion

This exploratory study estimated the relationship between population and area measures of county unincorporation with county population life expectancy and premature death for all counties in five U.S. states. Although bivariate associations demonstrated a relationship between county unincorporation measures and county health outcomes, when controlling for county economic, demographic, and structural factors, only the proportion of unincorporated surface area within the county was related to a county's average life expectancy. Unincorporated surface area may be associated with lower county health outcomes for several reasons. Larger proportion of a county's area that is unincorporated areas may be indicative of greater population sprawl, topographical challenges, and a lower revenue tax base that can impact the county government's ability to respond and provide services (Mattson, 2020; Mohr et al., 2010; Munroe, 2010; Purifoy, 2021). Populations living in counties with large unincorporated land that are more frontier or topographically challenging (e.g., mountainous, rugged, desert) may be hard to reach or have wide scatter between populations. This can increase a local government's costs due to economies of scale and access barriers when providing adequate infrastructure for water and sewer services (Mohr, Deller, & Halstead, 2010, Munroe, 2010) or transportation (Lockwood, 2004, Ravetz, Fertner, & Nielsen, 2012). Higher unincorporated populations in a county may be indicative of more densely populated unincorporated areas, which is more cost-efficient and more accessible to serve for county governments. These findings contribute to existing county-level health disparities research and suggest next steps in refining and developing measures of unincorporation status that might model relationships between county rates of unincorporation and health outcomes. Three findings in this study offer ideas for future research.

First, many contextual factors were significantly related to health outcomes, indicating the critical role of health and non-health-related contextual factors. Notably, as the starting year of data sampled was 2019 and extended into 2021, the COVID-19 pandemic likely explains the negative relationship between year and life expectancy (Schöley et al., 2022; Schwandt et al., 2022; Woolf et al., 2021). Likewise, across the full sample, unemployment and average median household income predict premature death in ways we might expect (Bundy et al., 2023; Stringhini et al., 2017). These results reinforce that economic status matters beyond other county characteristics when examining county-level health outcomes. Population economic factors contribute to reduced life expectancy through multiple pathways, including access to opportunities, information, and resources needed to address health (Link & Phelan, 2010). Still, more research is required to understand how individual economic factors may reflect the political economy (Bambra et al., 2019), which impacts political determinants of unincorporated places and potentially the health of residents within a county. Unincorporation status, county government, and vital social determinants (i.e., socioeconomic status, residential segregation, and education) combine in unique and complex pathways to generate the health outcomes of a county's population in incorporated and unincorporated places. Further study is needed to understand how a population's economic condition impacts county government's ability to respond to a population's health needs.

Second, as in much geographic and policy research, these findings underscore how difficult it is to capture and account for the multitude of pathways and accumulation of environmental, community, familial, and individual factors that contribute to life expectancy and premature death at a county level. Understanding contextual and compositional differences between incorporated and unincorporated places and people within the same county is essential to clarifying these relationships and pathways. For example, more study is needed to identify the racial and socioeconomic demographics of unincorporated and incorporated populations to assess if processes of segregation known to be associated with poor health play a role in similar outcomes (Lichter et al., 2015). Across time, incorporated boundaries have been used to exclude or seclude populations by social location in the U.S., which can mask residential segregation patterns maintained through annexation and mobility practices (Anderson, 2008, 2010; Marsh et al., 2010; Molina, 2014, pp. 180–187).

This study's use of Theil's H Index segregation measure as an aggregate of the total county segregation measure may also obscure relationships between unincorporation and county health. A strength of Theil's H Index segregation measure is its ability to measure segregation differences by census block compared to census block groups, which allows for measuring relatively small areas if densely populated; however, census blocks are population-based, obscuring the impact for census blocks with populations of less than 5000. Further, census blocks are not sensitive to jurisdictional boundaries across incorporated and unincorporated areas (U.S. Census Bureau, 2011). Research that identifies socioeconomic, racial, and ethnic composition in unincorporated areas in contrast to incorporated places is needed. Further, measures of racial and economic residential segregation that account for dissimilarities between incorporated and unincorporated place populations may contribute to a more nuanced understanding of the social inequalities in counties that can impact health.

Third, identifying additional factors to better capture the political mechanisms by which unincorporation impacts health could contribute to a revised measure of unincorporation and include additional county-level factors. Identifying political factors that impact the resources and capacity available to a county to serve its jurisdiction relative to the size of its population and area is critical. For these reasons, incorporating county revenues and authority measures may be essential in future research. Additionally, understanding and accounting for counties' autonomy and responsibility in delivering human services may be necessary. In some states, such as California, counties have a great deal of financial and decision-making responsibility in delivering human services. Conversely, in states like Texas, the state can play a decisive role in directing, financing, and organizing some human service delivery with exceptions such as provisions for safety net healthcare across the whole state. States with limited county autonomy may see counties more strained to reach unincorporated areas, while more limited county revenues may contribute to more limited opportunities to deliver services (Graaf et al., 2016). Alternatively, greater county autonomy may alleviate those concerns and allow for more racial bias in bureaucratic discretion at the local level, disproportionately disadvantaging residents of color (Elias, 2013; Wright II et al., 2021).

### 5.2 Limitations and future research needs

These indicators offer an important step in expanding research on the relationship between unincorporation and health, yet they are blunt measures, with limitations in differentiating how county government is impacted by the policies and practices associated with meeting the needs of unincorporated areas and residents. Because incorporated boundaries change very slowly, this study assessed only an association between unincorporation and health outcomes. Increasing causal inference, through difference of difference designs or the use of lagged effects, would require at least twenty years of data to generate meaningful findings. Another potential limitation is the use of life expectancy with small area populations. Life expectancy measures become unreliable when area and mortality rates are relatively small. The CHRR data includes NCHS data for county life expectancy, the smallest geography estimation produced by the U.S. Census. NCHS uses demographic and statistical modeling tools to produce reliable calculations to account for small and missing data ((National Center for Health Statistics (U.S.), 2018). While the CHRR data already accounts for this by suppressing data for counties with populations less than 5,000, we cannot rule out the possibility that life expectancy estimates for smaller counties are overestimated. Prior research does indicate that life expectancy estimates for populations greater than 5000 can be used with reasonable confidence (Arias et al., 2018; Eayres & Williams, 2004). Future research should capitalize on data sources that allow for county-level examinations of health data over multiple decades (e.g., healthcare claims) to strengthen causal inference.

To more specifically capture the importance of the burdens placed on counties by populations and areas under their direct care (especially relative to their resources), future efforts may combine population and area metrics to create a measure of the unincorporated population per unincorporated square mile (unincorporated population index/unincorporated area index) or unincorporated population density. Further, it would be helpful to develop methods that would capture the spatial distributions of where populations in unincorporated areas live relative to incorporated areas, as the impacts of unincorporation would likely be different for populations living near incorporated areas compared to those living in areas far from cities or towns. Geospatial methods may provide additional approaches to capture this level of nuance in unincorporated populations, such as remote sensing that draws on satellite imagery to identify rooftops or other residential structures. Finally, as this study included data from only five states, testing these indicators for all 50 states will better assess their strength as a predictor of health outcomes.

### 5.3 Conclusion

These findings demonstrate the continued need to investigate how unincorporation impacts county governance and population health. This research provides a foundation for exploring how unincorporated areas and populations are factors that should be considered in future health research. Policymakers, communities, constituents, and researchers using political indicators such as unincorporation to examine county health may further explore health inequities across places often marginalized in governance and research. As representative local government bodies and service providers, counties will continue to play critical roles in helping residents address short- and long-term health threats across the life course.

## CRediT authorship contribution statement

**Cristina Gomez-Vidal:** Writing – review & editing, Writing – original draft, Methodology, Investigation, Funding acquisition, Conceptualization. **Ashley N. Palmer:** Writing – review & editing, Writing – original draft, Methodology, Formal analysis. **Katherine Kitchens:** Writing – review & editing, Writing – original draft, Data curation. **G. Allen Ratliff:** Writing – review & editing, Methodology, Investigation, Funding acquisition, Conceptualization. **Genevieve Graaf:** Writing – review & editing, Visualization, Supervision, Project administration, Methodology, Investigation, Funding acquisition, Conceptualization.

## Ethical statement

The study used publicly available secondary data and did not involve human or animal subjects. The study was exempt from ethical approval per the University of Texas at Arlington's Institutional Review Board policies.

## Declaration of competing interest

The authors declare the following financial interests/personal relationships which may be considered as potential competing interests: Cristina Gomez-Vidal, Ashley Palmer, Katherine Kitchens, Allen Ratliff, and Genevieve Graaf report financial support was provided by County Health Rankings and Roadmaps program, a program of the University of Wisconsin Population Health Institute, with support from the Robert Wood Johnson Foundation. Funders did not have editorial review or approval of this work. The views expressed are those of the authors and do not necessarily reflect the views of County Health Rankings and Roadmaps or the Robert Wood Johnson Foundation.

## Data Availability

Data will be made available on request.

## Footnotes

2 In 2023, population growth in cities over 50,000 varied from 0.1% In the Midwest to 1.0% in the South. Of all incorporated cities, 75% have populations less than 5,000, decreased in population in the Midwest and Northeast from 0.3% to 0.1% and grew in the West by 0.3% and in the South by 0.6% (U.S. Census, 2023).

3 It is important to note that “unincorporated” and “rural” are separate concepts. There are unincorporated areas that are urban with high population densities (e.g., East Los Angeles and Kensington, CA), and there are municipal governments in rural communities throughout the U.S. While it is the case that most unincorporated areas are rural areas, they are distinctly different political and social concepts and should not be confused.

4 Appendix B provides a description of Table 1 variables.
