# Supplementary material for: Unincorporation in counties as a political determinant of health: An exploration of five states
Source: SSM Popul Health. 2024 Nov 19;28:101728. doi: 10.1016/j.ssmph.2024.101728 (PMC11647457; doi:10.1016/j.ssmph.2024.101728)
Supplement: Multimedia component 1 [file mmc1.docx]

**Appendix A. County Health Rankings Data Sources**

|  |  | **Data Source Years** | | |
| --- | --- | --- | --- | --- |
| **Variable** | **County Health Rankings and Roadmaps Data Source** | **2019** | **2020** | **2021** |
| Left Expectancy | National Center for Health Statistics - Mortality Files | 2015-2017 | 2016-2018 | 2017-2019 |
| Premature Death | National Center for Health Statistics - Mortality Files | 2015-2017 | 2016-2018 | 2017-2019 |
| Rural population - Census | U.S. Census Bureau | 2010 | 2010 | 2010 |
| High school completion | EDFacts | 2017-2018 | 2017-2018 | 2017-2018 |
| Unemployment rate | Bureau of Labor Statistics | 2017 | 2018 | 2019 |
| Median household income | Small Area Income and Poverty Estimates | 2017 | 2018 | 2019 |
| Population under age 65 without health insurance | Small Area Income and Poverty Estimates | 2017 | 2018 | 2019 |
| County proportion of state population | U.S. Census Bureau | 2019 | 2019 | 2019 |
| County proportion of state area | U.S. Census Bureau | 2019 | 2019 | 2019 |
|  |  |  |  |  |
