# Supplementary material for: Unincorporation in counties as a political determinant of health: An exploration of five states
Source: SSM Popul Health. 2024 Nov 19;28:101728. doi: 10.1016/j.ssmph.2024.101728 (PMC11647457; doi:10.1016/j.ssmph.2024.101728)
Supplement: Multimedia component 2 [file mmc2.docx]

| **Appendix B. Table 1 Variable Description** | |
| --- | --- |
| **Variables** | **Description** |
| Population | Proportion of unincorporated population in a county |
| Area | Proportion of unincorporated area in a county |
| Life expectancy | Average number of years from birth that one may expect to live (age-adjusted) |
| Premature death | Years of potential life lost before age 75 per 100,000 population (age-adjusted) |
| PCP ratio (per 1000 persons) | Rate or ratio primary care providers to populations (per 1,000 persons) |
| Access to mental health providers | Rate or ratio of population to mental health providers |
| Uninsured | Percentage of population under age 65 without health insurance. |
| High school completion | Percentage of adults ages 25 and over with a high school diploma or equivalent. |
| High school completion | American Community Survey, 5-year estimates 2015-2019 |
| Rural population | Percentage of county population living in a rural area |
| Residential segregation | Theil-H index measures level of racial segregation by measuring the evenness of distribution of diversity across multiple racial/ethnic categories within a county |
| Racial group population | Percentage Non-Hispanic Black, American Indian & Alaska Native, Asian, Native Hawaiian/Other Pacific Islander, non-Hispanic White |
| High school completion | Percentage of adults ages 25 and over with a high school diploma or equivalent. |
| Unemployment rate | Percentage of population ages 16+ unemployed but seeking work |
| Median household income | The income where half of households in a county earn more and half of households earn less. |
| Uninsured porportion | Percentage of population under age 65 without health insurance. |
| PCP ratio (per 1000 persons) | Rate or ratio primary care providers to populations (per 1,000 persons) |
| County area proportion | Proportion of county surface area relative to total statesurface area |
| County population proportion | Proportion of county population relate to state total popoulation |
